# Gut Microbiota: Implications in Alzheimer’s Disease

**DOI:** 10.3390/jcm9072042

**Published:** 2020-06-29

**Authors:** Yixi He, Binyin Li, Dingya Sun, Shengdi Chen

**Affiliations:** 1 Department of Neurology and Institute of Neurology, Ruijin Hospital affiliated to Shanghai Jiao Tong University School of Medicine, Shanghai 200025, China; heyixiheyixi@163.com (Y.H.); libinyin@126.com (B.L.); 2 Institute of Neuroscience, Key Laboratory of Molecular Neurobiology of the Ministry of Education, Second Military Medical University, Shanghai 200433, China; dingyasun@163.com

**Keywords:** Alzheimer’s disease, gut microbiota, microbiota–gut–brain axis, neurodegenerative disease, intestinal flora

## Abstract

Alzheimer’s disease (AD), the most common cause of dementia, is a neurodegenerative disease that seriously threatens human health and life quality. The main pathological features of AD include the widespread deposition of amyloid-beta and neurofibrillary tangles in the brain. So far, the pathogenesis of AD remains elusive, and no radical treatment has been developed. In recent years, mounting evidence has shown that there is a bidirectional interaction between the gut and brain, known as the brain–gut axis, and that the intestinal microbiota are closely related to the occurrence and development of neurodegenerative diseases. In this review, we will summarize the laboratory and clinical evidence of the correlation between intestinal flora and AD, discuss its possible role in the pathogenesis, and prospect its applications in the diagnosis and treatment of AD.

## 1. Introduction

Alzheimer’s disease (AD), a neurodegenerative disease, as well as the most common cause of dementia in the elderly population, is a serious threat to human health and quality of life. Currently, there are 30 million cases of AD worldwide, and the number is expected to rise to 90 million by 2050 as the population ages [1]. China has the largest population of dementia patients in the world, which brings great pressure to its society [2]. AD usually has three stages: preclinical, mild cognitive impairment and dementia. Mild cognitive impairment, characterized mainly by forgetting, is the most common and most likely to develop into dementia. The pathological manifestations of AD mainly include extensive plaques formed by extracellular amyloid beta (Aβ) aggregation and intracellular neurofibrillary tangles formed by over-phosphorylated TAU protein, which are the gold standard for the pathological diagnosis of AD [3,4,5]. However, the deposits of Aβ and Tau have been shown to appear in the brain 10–20 years before the onset of clinical symptoms of dementia [3]. As for the clinical diagnosis of AD, although these biomarkers can be detected by positron emission tomography for Pittsburgh compound B (PiB) or lumbar puncture for CSF biochemical tests, few can accept radioactive or invasive testing when the patient is in very mild cognitive dysfunction, and diagnosing mainly depends on the evaluation of clinical symptoms. In terms of treatment, there is still a lack of effective drugs that can change the pathological process of AD despite the huge investment made by the industry [6]. Therefore, it is necessary to further clarify the pathological mechanism of AD, explore novel non-traumatic early biomarkers and develop new effective treatment strategies.

There are billions of microbes colonizing the human gut. Growing evidences suggest that there is a bidirectional connection between the gut microbiota and the brain, which is called the Microbiota–Gut–Brain Axis [7]. Gut dysbiosis has been revealed to be associated with neurodegenerative diseases, such as Parkinson’s disease and Huntington’s disease [8,9,10]. Currently, accumulating evidence supports the hypothesis that gut microbiota are closely related to the occurrence and development of many neurological diseases [7,11]. Experimental studies have shown that intestinal flora regulate brain functions such as learning and memory [12]. The intestinal microbiota can secrete a large amount of amyloids and lipopolysaccharide, which may lead to changes in signaling pathways and the production of pro-inflammatory factors, leading to Aβ deposition [13]. More importantly, the composition and function of gut microbiota can affect the pathophysiology of age-related cognitive impairment and dementia, suggesting that changes of intestinal flora may be one of the causes of AD [14,15,16,17]. Despite the fact that many excellent reviews have discussed the associations between gut microbiota and their role in AD and other neurodegenerative disorders [18,19,20,21,22], an in-depth comprehensive literature revision on the gut microbiota implications in AD is still greatly needed in order to understand the pathogenesis and to develop possible therapeutic and diagnostic strategies. Here, we intend to summarize gut microbiota alterations in AD patients and discuss their possible roles in the pathogenesis, and prospect its clinical implications.

## 2. Gut Microbiota Alterations in AD Patients

The studies of gut microbiota alterations in AD patients are summarized in Table 1. Analysis of intestinal flora in patients with AD-related cognitive dysfunction was first conducted by Cattaneo A and his colleagues [23]. In patients with cognitive impairment and brain amyloidosis (*n* = 40), patients with cognitive impairment without brain amyloidosis (*n* = 33) and healthy controls (*n* = 10), they measured the abundance of selected bacterial gut microbiota taxa in the feces, as well as the levels of cytokines and inflammatory factors in the blood. The characteristics of AD patients and healthy controls were of no significant difference regarding age, gender and body mass index. They found an increased abundance of the pro-inflammatory species Escherichia/Shigella in the intestinal flora of people with cognitive impairment and brain amyloidosis, while the abundance of anti-inflammatory species E. Rectale decreased. Moreover, the levels of pro-inflammatory cytokines IL-1β, NLRP3 and CXCL2 were positively correlated with inflammatory Escherichia/Shigella abundance and negatively correlated with anti-inflammatory E. Rectale. Haran JP and his colleagues recruited AD patients (*n* = 24, with average age 84.7 years) and gender-matched controls (*n* = 51, with average age 83.0 years), in which there were no significant difference in age, gender or medical history between the groups. They showed that the proportion of bacteria capable of synthesizing butyrate in the microbiota of AD patients’ feces decreased, while the abundance of pro-inflammatory bacteria increased [24]. They also found that fecal samples from elderly patients with AD could induce lower expression level of p-glycoprotein (a key mediator of intestinal homeostasis) in intestinal epithelial cells in vitro, and p-glycoprotein dysregulation would contribute directly to inflammatory disorders of the intestine. These studies suggest that disturbance in the gut microbiota, which leads to loss of intestinal homeostasis and inflammation, may underlie neurodegenerative disease.

Vogt et al. examined the composition of intestinal microbiota of AD patients (*n* = 25) and controls (*n* = 25). The characteristics of patients with AD and healthy controls were of no significant differences in age, gender, ethnicity, body mass index or diabetes status [25]. The results showed that the intestinal microbial diversity decreased in AD patients. Compared with the control group, the abundance of Firmicutes and Actinobacteria in AD patients was lower, while the abundance of Bacteroidetes was higher. They also analyzed the correlation between the levels of genera abundance and the cerebrospinal fluid biomarkers for AD, and suggested that AD should be included in the list of diseases associated with microbial changes in the gut. Zhuang ZQ et al. collected fecal samples from AD patients (*n* = 43) and normal controls (*n* = 43), and analyzed the fecal flora composition [26]. There was no significant difference in education and comorbidities of hypertension, diabetes mellitus, hypercholesterolemia and coronary heart disease between AD patients and normal controls. They found that several bacteria taxa in AD patients were changed at taxonomic levels. They further demonstrated that the decreased abundance of Bacteroides and Lachnoclostridium at genus level may represent the alterations at the family level in the intestinal bacteria of AD patients.

Cognitive dysfunction is the clinical characteristic of AD. Although studies have suggested changes in the intestinal flora of AD, it is unclear whether these changes are related to patients’ cognitive dysfunction. Liu P et al. recruited a total of 97 subjects (33 AD, 32 aMCI and 32 healthy controls), in which all were right-handed, aged 50 to 85 years, with at least six years of education. They found a decrease in fecal microbial diversity and significant differences in microbial composition in AD patients compared to aMCI patients and healthy controls [27]. Compared with the control group, in the feces of AD patients, the proportion of Firmicutes decreased significantly, while the abundance of Proteobacteria increased. Gammaproteobacteria, Enterobacteriales and Enterobacteriaceae were shown to have an enriched prevalence from aMCI to AD patients. There was a significant correlation between clinical severity score and changes in microflora abundance in patients with AD. In our study, a total of 90 subjects (30 AD, 30 aMCI and 30 healthy controls) were recruited, and the characteristics of patients with AD and MCI and healthy controls were of no significant differences in age, gender, education, body mass index or constipation. The mini-mental State Examination (MMSE) scale was evaluated, and the composition of gut and blood bacterial communities was determined by 16S ribosomal RNA gene sequencing [28]. Our results showed that patients with AD have similar intestinal flora to those with MCI. Compared with the control group, the abundance of Alistipes, Bacteroides, Parabacteroides, Sutterella and Paraprevotella in the fecal samples of AD patients was decreased. Notably, the reduction of Bacteroides has been reported in another report [26], which suggests that this genus may be a protective factor of AD. The proportion of Dorea, Lactobacillus, Streptococcus, Escherichia, Bifidobacterium and Blautia in the fecal samples of AD patients was increased, among which the increased abundance of Blautia and Dorea was correlated with a decreased MMSE score, suggesting that they were risk factors for the development of AD. The abundance of Escherichia in the blood and stool samples of AD and MCI patients was increased, suggesting that Escherichia may be involved in the pathogenesis of AD, given that the deposition of Aβ in the brain of AD patients may be associated with central bacterial infection and inflammation.

## 3. The Pathogenic Role of Gut Microbiota in AD

### 3.1. Gut Microbiota Alterations in AD Models

**Figure 1:**
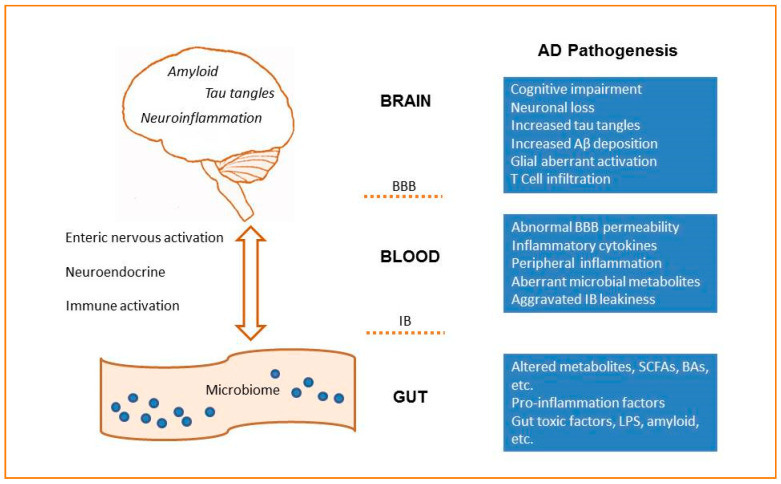
The microbiota–gut–brain axis in AD. The microbiota–gut–brain axis is a bidirectional communication system that includes neural, immune, endocrine and metabolic pathways. Dysbiosis of gut microbiota would affect the permeability of the intestinal barrier (IB), induce the leak of the brain–blood–barrier (BBB), and aggravate neuroinflammation and AD pathologies.

Many studies have shown that changes in intestinal flora composition lead to increased intestinal barrier permeability and systemic inflammation, which impairs the blood–brain barrier, promotes neuroinflammation, and ultimately results in neurodegeneration (Figure 1). It is proposed that the alterations in the network composed of intestinal flora, mucosal immune system and enteric nervous system could represent a common path driving the onset of neurodegenerative diseases [29,30,31].

Many studies have shown significant alterations in gut microbiota in AD mouse models (Table 2). Peng W et al. compared the intestinal flora composition of AD model SAMP8 mice and control SAMR1 mice by 16S rRNA gene and metagenomic sequencing analysis. The results showed that the characteristic composition of intestinal microbiota of SAMP8 mice was significantly different from that of SAMR1 mice. Network analysis showed that the correlation density and clustering of operational taxonomic units in the intestinal flora of SAMP8 mice decreased [32]. Importantly, they observed that the abundance of *norank f Lachnospiraceae* and *unclassified f Lachnospiraceae* were increased in SAMP8 mice, which is consistent with a report showing the upregulation of *Lachnospiraceae* in AD patients [25]. Moreover, the abundance of *Alistipes* increased in SAMP8 mice, which have also been found to be more abundant in AD patients [25]. These consistent alterations in patients and animal models suggest the importance of theses taxa on AD pathogenesis. Zhan G. et al. found that 27 intestinal bacteria at 6 phylogenetic levels in SAMP8 mice were different from those in SAMR1 mice [33]. The pseudo germ-free mice showed significantly reduced cognitive function, and they showed improved behavioral and α, β diversity indices after being transplanted with the fecal bacteria of SAMR1 mice instead of SAMP8 mice. These results suggest that cognitive dysfunction in SAMP8 mice is associated with the abnormal composition of intestinal flora. Studies by Shen L. et al. on APP/PS1 transgenic mice, another AD mouse model, showed that the mice at 6 months had spatial learning and memory impairment, which was further worsened at 8 months [34]. They found that with the growth of age, the microbial diversity of APP/PS1 mice decreased. The abundance of Helicobacaceae and Desulfovibrionaceae at the family level, and Odoribacter and Helicobacter at the genus level were significantly increased in APP/PS1 mice, while the abundance of Prevotella was significantly decreased [34]. Interestingly, *Prevotellaceae* showed increasing abundance from 3.05% at 3 months old, 5.03% at 6 months old, to 10.30% at 8 months old, suggesting the association of this bacterial taxon with aging in APP/PS1 mice. Some studies on AD mouse models have shown increased levels of pro-inflammatory bacteria in the intestinal flora. For example, Bauerl C et al. compared the intestinal flora of APP/PS1 mice with their wild-type littermates. They found that the bacterial profiles were similar at 3 months, but the populations were different at 6 months. As age increases, Turicibacteriaceae and Rikenellaceae increased in all groups, although total Bacteroidetes remained stable. Proteobacteria, especially of the genus Sutterella, were specifically increased at 6 months in APP/PS1 mice. Moreover, the inflammation related family Erysipelotrichaceae was more abundant in APP/PS1 mice at 24 months, which suggested that AD pathology in mice shifts the gut microbiota towards profiles like inflammatory disorders [35].

### 3.2. Gut Microbiota and Neuroinflammation

Intestinal flora is closely associated with neuroinflammation, and this is crucial for the pathogenesis of AD [36,37]. Gram-negative facultative anaerobe Bacteroides fragilis constitutes a considerable proportion of the microbial community in the human gastrointestinal tract. Like most gram-negative bacilli, Bacteroides fragilis secretes an unusual mixture of neurotoxins, including pro-inflammatory lipopolysaccharides (LPS). Zhao Y et al. reported that the level of LPS of AD patients increased by two times in the neocortex and three times in the hippocampus compared with the control group of the same age, and LPS mainly existed around the nucleus [38,39]. Notably, Bacteroides fragilis and the neurotropic herpes simplex virus 1 share a common final pathway after activating NF-κB (p50/p65) [40,41]. Using the co-culture system of human neuronal-glial cells, Zhao Y et al. found that when lipopolysaccharide BF-LPS was added, presynaptic neurexin-1 (NRXN-1), synaptosomal-associated phosphoprotein-25 (SNAP-25), phosphoprotein synapsin-2 (SYN-2), postsynaptic protein neuroligin (NLGN), the sh3-ankyrin repeat domain and proline-rich cytoskeletal scaffolding protein (SHANK3) were all significantly down-regulated [42]. Moreover, the DNA transcription products in the culture cells were reduced when stimulated by LPS [43]. These studies suggest that pro-inflammatory toxins secreted by gastrointestinal bacteria may “leak” into the circulation and then enter the brain, causing neuroinflammation and affecting neuronal function.

Using the 5XFAD AD mouse model, Wang X et al. found that intestinal flora composition changed and led to the peripheral accumulation of phenylalanine and isoleucine, which further stimulated the differentiation and proliferation of pro inflammatory T helper 1 (Th1) cells [44]. The peripheral Th1 cells infiltrating into the brain are associated with microglia M1 activation, which lead to neuroinflammation. They also found that the levels of phenylalanine and isoleucine and the quantity of Th1 cells were elevated in the blood of patients with mild cognitive impairment. These results suggest that neuroinflammation caused by metabolic abnormalities due to intestinal flora imbalance may be associated with AD. Inflammation of the brain is not always a response to local primary insults in the periphery. However, by using a drosophila AD model, Wu SC et al. demonstrated that intestinal bacterial infection can stimulate immune cell recruitment into the brain, thus aggravating the progress of AD. This work emphasized the importance of gut–brain crosstalk in neurodegenerative diseases [45]. Minter MR et al. found that long-term combinatorial broad-spectrum antibiotic treatment would change the composition and diversity of gut microbes in APPSWE/PS1ΔE9 mice, reduce Aβ plaque deposits and attenuate glial activation, suggesting the diversity of gut microbiota can regulate the host’s innate immunity and affect Aβ amyloidosis [46].

### 3.3. Intestinal Metabolites

Changes in intestinal flora can cause alterations in intestinal metabolites. By using stable isotope labeling combined with liquid chromatography-tandem mass spectrometry, Zheng J et al. found that the contents of nine short-chain fatty acids (SCFAs) were significantly different between AD and wild type mouse fecal samples [47]. The level of propionic acid, isobutyric acid, 3-hydroxy butyric acid and 3-hydroxy isopropyl acid were reduced, while lactic acid, 2-hydroxy butyric acid, 2-hydroxy isobutyric acid, levulinic acid and valproic acid were increased. SCFAs are metabolites produced mainly by the intestinal flora from undigested fibers and proteins. As these molecules can act as G-protein-coupled receptor activators and histone deacetylase inhibitors, the changes of SCFAs in the feces of AD mice may be associated with the pathology of AD [47]. SCFAs have been shown to be able to inhibit the aggregation of Aβ in vitro [48]. Zhang L et al. compared the fecal microbiota and fecal SCFAs composition of wild-type and AD mice at different ages [49]. The results showed that the composition and diversity of the microbiota in AD mice were disturbed, and SCFAs levels were decreased, which may be related to the amyloid deposition and ultrastructural abnormalities in AD mouse intestines [49]. Tran TTT studied the relationship between the apolipoprotein E gene and gut microbes using APOE-targeted replacement (TR) transgenic mice [50]. They found the proportion of Prevotellaceae, Ruminococcaceae and several butyrate-producing genera changed. Metabolomics analysis of mouse feces revealed significant differences in microbial-related amino acids and short-chain fatty acids, suggesting that the most common genetic risk factor for AD is associated with specific intestinal microbiota profiles and metabolites changes. Yuan BF et al. synthesized stable isotopic labeling reagents to label metabolites with different chemical groups, including carboxyl, carbonyl, amine and sulfhydryl, and then combined these with liquid chromatography-mass spectrometry to analyze the mouse fecal metabolomics, and established the database of mouse fecal metabolomics [51]. They found significant differences in 211 fecal metabolites between AD and wild-type mice. It would be important to determine whether these metabolites are also changed in the feces of AD patients, as they could be new biomarkers for the diagnosis of AD. Trimethylamine N-oxide (TMAO) is a small molecule produced by the metabolism of dietary choline. CSF TMAO was shown to be higher in individuals with MCI and AD compared to healthy controls, and the elevated CSF TMAO was associated with AD pathological markers phosphorylated tau and Aβ42 [52]. Bile acids (BAs) are the end products of human cholesterol metabolism, which is produced by human and gut microbiota co-metabolism. In a 1562 case study, Nho K measured 20 kinds of primary and secondary BAs metabolites at the serum level, which were found to be related with amyloid proteins and tau in the cerebrospinal fluid of AD patients, as well as with brain atrophy and cerebral glucose metabolism dysfunction, thus providing further support for the role of this pathway in AD pathophysiology [53].

## 4. Clinical Implications of Gut Microbiota in AD

A large number of studies have shown that the composition and functionality of intestinal microbiota can affect the pathophysiology of age-related cognitive impairment and dementia, so it is highly possible to use fecal microbiota-related parameters and microbiota-derived metabolites as biomarkers for these cognitive-related diseases [16]. Based on the 20 kinds of typical predominant genera model, Liu P et al. could effectively distinguish AD and MCI from healthy controls [27]. They also found that the models based on the abundance of the Enterobacteriaceae family could distinguish AD from both MCI and healthy controls. Our research also showed that 93% of MCI patients could be distinguished by established random forest models with all different fecal genera [28]. These studies have facilitated the possibilities of finding clinically specific biomarkers for AD. However, gut microbes can be influenced by many factors, such as living region and diet. Although much evidence has been found supporting a close relationship between AD and gut microbes, no specific intestinal flora have been identified in AD patients; hence, large-scale sample screening and analysis is undoubtedly necessary.

Many studies have attempted to combat neurodegenerative disease by modifying the gut microbiota [11,54]. The studies to modify the gut microbiota in AD patients and models are summarized in Table 3. Amyloid and neurofibrillary tangles transgenic (ADLPAPT) mice show amyloid deposits, neurofibrillary tangles, reactive gliosis and memory defects. Kim MS et al. found that fecal transplantation from wild-type mice to ADLPAPT mice reduced the formation of amyloid plaques and neuronal tangles, and alleviated glial responses and cognitive impairment [55]. Sun J et al. used APPswe/PS1dE9 transgenic mice, another AD model, and found that fecal microbiota transplantation alleviated the cognitive decline and decreased the deposition of Aβ in the brain [56]. Further analysis showed tau phosphorylation, and that the level of Aβ40 and Aβ42, and COX-2 or CD11b positive cells decreased, while synaptic plasticity increased, indicating an overall improved pathological condition.

Bifidobacterium Iongum (NK46), a probiotic isolated from human intestinal flora, was found to have a strong inhibitory effect on the production of intestinal microbial endotoxin and the activation of NF-κB in BV-2 cells after LPS stimulation [57]. Using 5xFAD-Tg mice, an AD model, they demonstrated that oral administration of NK46 could change the composition of gut microbiota, especially the proportion of Proteobacteria, reduce the level of LPS in the feces and blood, and increase the colon tight junction protein expression. Moreover, administration of NK46 inhibited amyloid-β, β/γ-secretase and caspase-3 expression in the hippocampus, and alleviated the cognitive decline [57]. Kobayashi Y et al. studied the effects of oral administration of Bifidobacterium breve strain A1 (B. breve A1) on the behavior and pathophysiological process of mice injected with Aβ25-35 in the lateral ventricle [58]. They found that B. breve A1 reversed the impairment on alternate behavior in a Y maze experiment, and reduced the latency in a passive avoidance experiment. Gene expression profile analysis showed that B. breve A1 could suppress the expressions of inflammationrelated genes induced by Aβ in the hippocampus [58]. The mixed probiotics (Lactobacillus acidophilus, Bifidobacterium bifidum and Bifidobacterium longum) treatment was also found to improve the impaired spatial cognition ability and restore synaptic plasticity in the rats injected with Aβ in lateral ventricle [59]. Bonfili L et al. studied the role of a probiotic (SLAB51) in combating AD-associated oxidative brain damage [60]. By using a 3xTg-AD model mouse, they found that SLAB51 affected the composition of intestinal flora and its metabolites, as well as plasma concentrations of inflammatory cytokines and key metabolic hormones. They observed a partial recovery of damaged neuronal proteolytic pathways and less amyloid accumulation; thus, the cognitive impairment was alleviated [60]. They also found that administration of SLAB51 significantly reduced oxidative stress in the brains of AD mice by activating sirt1-dependent mechanisms [61]. Hoffman JD et al. reported that inulin, a non-digestible carbohydrate fiber fermented in the gastrointestinal tract, could increase the beneficial microbiota, reduce the harmful microbiota, and enhance peripheral and brain metabolism in APOE4 transgenic mice. Inulin also reduced inflammatory gene expression in the hippocampus, suggesting that a prebiotic diet may help reduce the risk of AD in asymptomatic APOE4 carriers [62]. Westfall S et al. examined the effects of synbiotic formula (containing three metabolically active probiotics and a polyphenol-rich prebiotic) on an AD drosophila model, and they found that synbiotic treatment could improve the animal survival rate and mobility, and reverse amyloid deposition and acetyl cholinesterase activity [63]. Taken together, these findings suggest that a diet supplement of probiotics may provide a promising adjuvant therapy for AD.

Dietary habits are closely related to the composition of intestinal bacteria. A ketogenic diet has been reported to increase the abundance of intestinal beneficial taxa (Akkermansia muciniphila and Lactobacillus) and reduce the abundance of intestinal pro-inflammatory taxa (Desulfovibrio and Turicibacter) [64]. It also increased the cerebral blood flow and p-glycoprotein transports on the blood–brain barrier to facilitate the clearance of Aβ, suggesting that a ketogenic diet may reduce the risk of AD. Nagpal R et al. compared the differences of intestinal flora between elderly people with mild cognitive impairment and elderly people with normal cognition, and found that, in MCI subjects, Proteobacteria was positively correlated with the ratio of Aβ1-42/Aβ1-40, and fecal propionate and butyrate were negatively correlated with Aβ1-42. After adopting the mediterranean-ketogenic diet (MMKD), the abundance of Enterobacteriaceae, Akkermansia, Slackia, Christensenellaceae and Erysipelotriaceae increased, while the abundance of Bifidobacterium and Lachnobacterium decreased. MMKD also increased the level of propionate and butyrate while slightly decreasing the level of lactic acid and acetate in feces. These results suggest that a ketogenic diet regulates intestinal microbiota and metabolites, and is associated with improvement of AD pathology [65]. 

Some compounds derived from plants with anti-AD effects were found to regulate intestinal flora in animal studies. Silymarin and its main active component silybin administration could alleviate the memory deficits and reduce amyloid plaques in APP/PS1 mice [15]. Meanwhile, the diversity of the bacterial community decreased, and the abundance of several key bacterial species associated with AD changed, suggesting that the regulation of gut microbiota is involved in their effects against AD [15]. Pomegranate extract administration significantly reduced the expression of inflammatory biomarkers in the hippocampus of transgenic AD R1.40 mice [66]. Yuan et al. found that urolithin (6H-dibenzo [b,d]pyran-6-one derivatives), an intestinal metabolite of ellagitannins from pomegranate, which can pass through the blood–brain barrier, could prevent fibrosis of Aβ in vitro. Urolithin significantly reduced the levels of nitric oxide, interleukin 6, prostaglandin E2 and tumor necrosis factor in LPS-stimulated BV-2 cells. Furthermore, Methyl-urolithin B (3-methoxy-6h-dibenzo [B, d] pyran-6-one) had protective effects on the neurotoxicity and paralysis of Caenorhabditis induced by Aβ1-42 [67]. This result indicates that the intestinal metabolites derived from plant elements may have protective effects against AD.

There is emerging evidence that oligosaccharides could regulate the intestinal microflora, which may be developed as drugs to treat AD. Sun et al. examined the effect of fructooligosaccharides (FOS) on AD using Apse/PSEN 1dE9 (APP/PS1) mice, and found that treatment with FOS for 6 weeks reversed the changes in intestinal microbial composition and alleviated cognitive dysfunction and pathological changes. FOS significantly up-regulated the expression levels of synapsin 1 and PSD-95 whilst decreasing the phosphorylation level of JNK [68]. Oligosaccharide (OMO) is an oligosaccharide derived from Morinda officinalis. OMO was shown to improve memory behavior, reduce neuronal cell apoptosis and down-regulate the expression of Aβ 1-42 in both APP/PS1 transgenic mice and Aβ1-42-induced deficient rats [69,70].The CA-30, an oligosaccharide fraction derived from Liuwei Dihuang decoction, was shown to be able to modulate intestinal microbiota, rebalance the gut microbe-neuroendocrine immune regulatory network and ameliorate the cognitive deterioration of SAMP8 mice [71,72]. By using a 5XFAD AD mouse model, Wang X et al. found that GV-971, a sodium oligomannate, could inhibit peripheral phenylalanine/isolucine accumulation, control neuroinflammation and reverse cognitive impairment [44].

## 5. Future Perspectives

Although there is growing evidence supporting the close relationship between the intestinal flora and cognitive dysfunction in AD patients, more cautious evaluation and analysis of the data are greatly needed. Firstly, it is necessary to collect samples from patients on a large scale and in multiple centers, as gut microbiota are easily affected by patient’s location, diet, living habits and gastrointestinal tract infection. For instance, the reduced abundance of Bacteroides was found in the fecal samples of AD patients from Chinese cohorts [26,28]. On the contrary, intestinal Bacteroides have been reported to be increased in AD patients from the USA [24,25]. In addition, we found a higher abundance of *Blautia* in AD patients [28], which is consistent with a previous report [25]. However, another study showed that the relative abundance of *Blautia* was dramatically decreased [27]. These inconsistent results warrant further validation and investigation. Secondly, the diagnosis standardization of AD needs to be strengthened. Beside cognitive scale assessment, CSF biomarker analysis and brain imaging are required. The most exciting part of intestinal flora analysis in AD patients is the possibility to provide a non-invasive, patient-compliant diagnostic approach. Therefore, the intestinal flora spectra of AD patients should be compared not only with healthy controls, but also with those of patients with other neurodegenerative diseases (such as PD, MSA, etc.). This could not only reveal the specificity of AD intestinal flora, but also provide new clues for its etiological research.

Currently, there are still no effective treatments for AD; thus, the strategy of regulating intestinal microbial flora to treat AD has attracted great attention. The probiotic preparation (Lactobacillus casei W56, Lactococcus lactis W19, Lactobacillus acidophilus W22, Bifidobacterium lactis W52, Lactobacillus paracasei W20, Lactobacillus plantarum W62 Bifidobacterium lactis W51, Bifidobacterium bifidum W23 and Lactobacillus salivarius W24) was tested in 20 AD patients for 4 weeks. Administration of the probiotics affected the composition of intestinal bacteria in AD patients, with an increased abundance of Faecalibacterium prausnitzii and decreased abundance of the inflammation-related bacteria. Since the study was only observed for 4 weeks, to avoid variability in repeated tests, they did not compare changes in cognitive scales [73,74]. However, after finishing a trial of 96 patients with severe AD treated with probiotics for 12 weeks, they concluded that the AD patients were insensitive to probiotics based on cognitive scale assessment and serum biomarker evaluation. [75]. Undoubtedly, more rigorous clinical trials are needed to assess the efficacy of this probiotic preparation on the treatment of AD patients. Recently, in a phase 3 clinical trial in China, GV-971 (a mixture of acidic oligosaccharides) was shown to improve cognition in AD patients by suppressing gut dysbiosis and the associated phenylalanine/isoleucine accumulation, and was conditionally approved as a therapeutic drug for AD [44]. However, large-scale, multicenter, prudent clinical efficacy tests are extremely necessary.

Many studies on animal models of AD have revealed changes in intestinal flora, providing evidence for the role of gut microbiota in the pathogenesis of AD. However, up to now, most of the experimental evidence has come from correlation research; many studies still remain to describe the alterations of intestinal flora, and rigorous sufficient and necessary proof is still lacking. The microbiota–gut–brain axis is a bidirectional communication system that includes neural, immune, endocrine and metabolic pathways (Figure 1). Dysbiosis of gut microbiota can affect the permeability of the intestine and the blood–brain barrier, which contributes to AD pathologies. On the other hand, the brain may also regulate the intestinal flora through vagus nerve activity and neuroendocrine. For example, it was found that Aβ was not only expressed in the brain, but also in the intestinal tissues in 5xFAD mice. Fecal protein analysis revealed reduced trypsin levels in this mouse model compared to wild-type mice [76]. Therefore, the pathological changes in the intestinal tract of AD mice may also result from the neurological function variation caused by Aβ abnormal expression. In addition, the secreted miRNAs are able to enter microbial organisms, and the host’s miRNA might affect the intestinal microbial ecosystem [77]. Therefore, it is likely that AD-related miRNAs may enter intestinal bacteria and produce diseased microbiota. It should be taken into consideration that changes in gut flora may be the result of AD rather than the cause, although in vivo validation is greatly needed.

The underlying mechanism of how microbiota affect host neuronal activity and behavior is a very interesting question. Recent studies by Chu C et al. have found that manipulation of the microbiota in antibiotic-treated or germ-free adult mice resulted in significant deficits in fear extinction learning, which was associated with defective learning-related remodeling of postsynaptic dendritic spines and decreased activity in cue-encoding neurons in the medial prefrontal cortex [12]. Further metabolomics analysis identified four metabolites that were significantly down-regulated in germ-free mice and have been reported to be associated with neuropsychiatric disorders in both mouse models and humans, suggesting that compounds derived from the microbiota may directly affect brain function and behavior. Certainly, it would be interesting to further examine the levels of these metabolites in the blood and CSF of AD patients. To examine whether microbiota from AD patients would affect animal behavior, Fujii Y et al. transplanted fecal samples from a healthy control and an AD patient to germ-free C57BL/6N mice [78]. They found that, compared with the former, the latter performed significantly worse on an object location test and an object recognition test at 55 weeks of age, suggesting that the intestinal microbiota transplanted from the patients do affect the behavior of the mice. Further studies with samples from more AD patients are greatly needed. Moreover, investigations to clarify the molecular mechanism underlying this phenomenon are undoubtedly helpful for understanding the etiology of AD.

In conclusion, accumulating evidence of the association between gut microbiota and AD has been revealed. Further study on the role of intestinal flora in the pathogenesis of AD may develop new strategies for the diagnosis and treatment of the disease.

## Figures and Tables

**Table 1:** Gut microbiota alterations in patients with Alzheimer’s disease.

Cases	Age	Clinical Evaluation	Alterations of Abundance at the Different Taxa Level					References
			Phylum	Class	Order	Family	Genus	
33	74.85 (11.37)	MMSE; MoCA	↑ Proteobacteria	↑ Gammaproteobacteria	↑ Enterobacteriales	↑ Enterobacteriaceae	/	Liu, et al. 2019
			↓ Firmicutes	↓ Clostridia	↓ Clostridiales	↓ Clostridiaceae, Lachnospiraceae, Ruminococcaceae	↓ Blautia, Ruminococcus	
30	66.3 (5.1)	MMSE; MTA	/	/	/	/	↑ Bifidobacterium, Blautia, Dorea, Escherichia, Lactobacillus, Streptococcus	Li, et al. 2019
			/	/	/	/	↓ Alistipes, Bacteroides, Parabacteroides, Paraprevotella, Sutterella	
24	84.7 (8.1)	CDR	/	/	/	/	↑ Alistipes, Bacteroides, Barnesiella, Collinsella, Odoribacter	Haran, et al. 2019
			/	/	/	/	↓ Eubacterium, Lachnoclostridium, Roseburia	
43	70.12 (8.78)	MMSE; ADL; CDR; PiB	↑ Actinobacteria	↑ Actinobacteria, Bacilli	↑ Lactobacillales	↑ Enterococcaceae, Lactobacillaceae, Ruminococcaceae	↑Subdoligranulum	Zhuang et al. 2018
			↓ Bacteroidetes	↓ Bacteroidia, Negativicutes	↓ Bacteroidales, Selenomonadales	↓ Bacteroidaceae, Lanchnospiraceae, Veillonellaceae	↓Bacteroides, Lachnoclostridium	
40	71 (7.0)	MMSE; Amyloid PET	/	/	/	/	↑ Escherichia/Shigella	Cattaneo, et al. 2017
			/	/	/	/	↓ Bacteroides fragilis, Eubacterium rectale	
25	71.3 (7.3)	CDR; CSF marker	↑ Bacteroidetes			↑ Bacteroidaceae, Rikenellaceae, Gemellaceae	↑ Alistipes, Bacteroides, Bilophila, Blautia, Gemella, Phascolarctobacterium	Vogt, et al. 2017
			↓ Firmicutes, Actinobacteria			↓ Ruminococcaceae, Bifdobacteriaceae, Clostridiaceae, Mogibacteriaceae, Turicibacteraceae, Peptostreptococcaceae	↓ Adlercreutzia, Bifdobacterium, cc115, Clostridium, Dialister, SMB53, Turicibacter	

Abbreviations: Age, years (SD); MMSE, Mini-Mental State Examination; ADL, Activities of Daily Living; CDR, Clinical Dementia Rating; MoCA, Montreal Cognitive Assessment; Amyloid PET, amyloid positron-emission tomography (PET) with 18 F-Florbetapir; PiB, Positron emission tomography for Pittsburgh compound B to detect and quantify Aβ deposition; MTA, Medial temporal atrophy score measured by structural MRI; CSF marker, CSF biochemical tests including Aβ42, Aβ40, phosphorylated tau. ↑, increased abundance; ↓, decreased abundance.

**Table 2:** Gut microbiota alterations in AD mice.

AD Model	Control	Age	Alterations of Abundance	References
APP/PS1	WT	3, 6 and 8 months	At 3, 6 and 8 months: ↑ *Helicobacteraceae, Desulfovibrionaceae, Odoribacter, Helicobacter*; ↓ *Prevotella*. At 6 and 8 months: ↑*Coriobacteriaceae*; ↓*Ruminococcus*.	Shen et al. 2017
APP/PS1	WT	6 and 24 months	At 6 and 24 months: ↑*Proteobacteria*, *Sutterella*; ↓*Rikenellaceae*. At 24 months: ↑*Erysilopelotrichaceae*.	Bauerl et al. 2018
5xFAD	WT	9 weeks	↑*Firmicutes*, *Clostridium leptum group*; ↓*Bacteroidetes*.	Brandscheid C. et al. 2017
SAMP8	SAMR1	7 months	↓*Deferribacteres, Deferribacteres, Deltaproteobacteria, Deferribacterales, Desulfovibrionales, Mollicutes RF9, Clostridiales vadinBB60 group, Desulfovibrionaceae, Christensenellaceae, Family XIII, Deferribacteraceae, Ruminococcaceae, Mucispirillum, Serratia, Family XIII AD3011 group, Christensenellaceae R-7 group, Subdoligranulum, Desulfovibrio, Ruminiclostridium 9, Coprococcus 1, Ruminococcaceae UCG 004, Lachnospiraceae NK4A136 group, Lachnospiraceae oscillibacter*	Zhan et al. 2018
SAMP8	SAMR1	8 months	↑*norank f Lachnospiraceae*, *Alistipes, Odoribacter*, *unclassified* f Lachnospiraceae, *Akkermansia* in SAMP8; ↑ *norank_f Bacteroidales S24-7 group, Prevotella 9, Parasutterella, Butyrivibrio* in SAMR1.	Peng et al. 2018

**Abbreviations** SAMP8: senescence accelerated mouse prone 8 mice; SAMR1: senescence-accelerated mouse resistant 1 mice; APP/PS1: APPswe/PS1dE9 mice; 5xFAD: 5xFAD (APP K670N, M671L, I716V, PS1 M146L, L286V) mice; WT: wild type; ↑, increased abundance; ↓, decreased abundance.

**Table 3:** Summary of studies to modify the gut microbiota in AD models and patients.

Intervention	Subjects	Main Effects	References
Faecal microbiota transfer	ADLPAPT	Reduction of the Aβ deposition, neurofibrillary tangle formation, and glial activation; reversed abnormalities in the colonic expression of genes related to inflammatory responses; ameliorated cognitive impairment.	Kim MS et al. 2019
Faecal microbiota transfer	APP/PS1	Reduction of the Aβ deposition and decreased phosphorylation of tau protein; reduced expression of COX-2 and CD11b, and increased expression of PSD-95 and synapsin I; improvement of cognitive deficits.	Sun J et al. 2019.
*Bifidobacterium longum*	5XFAD	Shifted gut microbiota composition and reduced fecal and blood LPS levels; suppressed NF-κB activation, decreased expression of TNF-α, and upregulated expression of tight junction protein in the colon; suppressed caspase-3 expression and Aβ accumulation in the hippocampus; alleviated cognitive decline.	Lee HJ et al. 2019
*Bifidobacterium breve strain A1*	Aβ treated mice	Suppressed hippocampal expressions of inflammation and immune-reactive genes; improved behavior in a Y maze test and the reduced latency time in a passive avoidance test.	Kobayashi Y et al. 2017
Mixed probiotics	Aβ treated rats	Improvement of the anti-oxidant/oxidant biomarkers; restored LTP and improved the maze navigation.	Rezae Asl Z et al. 2019
SLAB51	3xTg-AD	Shifted plasma concentration of inflammatory cytokines and key metabolic hormones; reduced oxidative stress and restoration of impaired neuronal proteolytic pathways; alleviated cognitive decline.	Bonfili L et al. 2017; Bonfili L et al. 2018
Inulin	E4FAD	Increased abundance of beneficial microbiota and reduced harmful microbiota in the feces; higher levels of SCFAs, tryptophan-derived metabolites, bile acids and glycolytic metabolites; suppressed the hippocampal expressions of inflammatory genes.	Hoffman JD et al. 2019
Symbiotic formulation	AD-drosophila	Rescued Aβ deposition and acetylcholinesterase activity; increased survivability and motility.	Westfall S et al. 2019
MMKD	MCI patients	Modified gut microbiota composition and metabolites; improved AD biomarkers in CSF.	Nagpal R et al. 2019
Silymarin and silibinin	APP/PS1	Regulative effect in abundances on bacterial species associated with AD development; reduce the amyloid plaque burden; alleviated memory deficits.	Shen L et al. 2019
Urolithins	AD-elegans	Suppressed Aβ fibrillation in vitro; protective effects against Aβ-induced neurotoxicity; increased the maximum survival/mobility.	Yuan T et al. 2016
Fructooligosaccharides	APP/PS1	Reversed the alteration of microbial composition; increased expression of glucagon-like peptide-1 in the gut; up-regulated expression of synapsin I and PSD-95.	Sun et al. 2019
OMO	APP/PS1	Regulative effect on the composition and metabolism of the gut microbiota; suppressed brain tissue swelling and neuronal apoptosis and downregulated expression of Aβ; ameliorated memory deficits.	Xin Y et al. 2018
OMO	Aβ treated rats	Regulated the composition and metabolism of gut microbiota; suppressed oxidative stress and inflammation; improvement of the learning and memory abilities.	Chen D et al. 2017
CA-30	SAMP8	Beneficial effects on the gastrointestinal microbiota dysbiosis; delayed aging processes; ameliorated cognitive impairments.	Wang J et al. 2016; Wang J et al. 2019.
Probiotic preparation	AD patients	Changes in the composition of intestinal bacteria, with the increased abundance of *Faecalibacterium prausnitzii* and decreased abundance of the inflammation-related bacteria; decline of fecal zonulin concentrations, and increased serum kynurenine concentrations.	Leblhuber F et al. 2018; Leblhuber F et al. 2019
Probiotic preparation	AD patients	No pronounced changes in scores of Test Your Memory and levels of serum biomarkers (TNF-α, IL-6, IL-10, TAC, GSH, NO, MDA, and 8-OHdG).	Agahi A et al. 2018
GV-971	AD patients; 5XFAD; APP/PS1	Suppress of gut dysbiosis; harnesses of blood phenylalanine/isoleucine accumulation and neuroinflammation; cognition improvement in a phase 3 clinical trial.	Wang X et al. 2019

Abbreviations ADLPAPT: AD-like pathology with amyloid and neurofibrillary tangles transgenic mice; AD-*drosophila*: *drosophila* expressing human BACE1 and the 695 amino acid isoform of human APP; AD-*elegans*: *Caenorhabditis elegans* treated with Aβ1–42; APP/PS1: APPswe/PS1dE9 mice; E4FAD: asymptomatic APOE4 transgenic mice; SAMP8: senescence accelerated mouse prone 8 mice; 3xTg-AD: 129-Psen1tm1Mpm Tg (APPSwe, tauP301L)1Lfa/J transgenic mice; 5XFAD: 5XFAD transgenic (Tg) mouse model. Probiotic formulation and supplements: CA-30 (an oligosaccharide fraction derived from Liuwei Dihuang decoction); GV-971(a mixture of oligosaccharides with the degree of polymerization from 2 to 10); Mixed probiotics (*Lactobacillus acidophilus, Bifidobacterium bifidum and Bifidobacterium longum*); MMKD (a modified Mediterranean-ketogenic diet); OMO (an oligosaccharide from Morinda officinalis); SLAB51 (lactic acid bacteria and bifidobacterial); Probiotic preparation (*Lactobacillus casei W56, Lactococcus lactis W19, Lactobacillus acidophilus W22, Bifidobacterium lactis W52, Lactobacillus paracasei W20, Lactobacillus plantarum W62 Bifidobacterium lactis W51, Bifidobacterium bifidum W23 and Lactobacillus salivarius W24*); Symbiotic formulation (*Lactobacillus plantarum*, *L*. *fermentum*, *Bifidobacteria longum spp. infantis* and a polyphenol rich polyphenol plant extract from the gastrointestinal tonic Triphala); Urolithins: gut microbiota-derived metabolites of pomegranate extract.
